# Gene Therapy for Cancer

**DOI:** 10.1038/sj.bjc.6604205

**Published:** 2008-02-05

**Authors:** S Schepelmann, C J Springer

**Affiliations:** 1 The Institute of Cancer Research, Cancer Research UK, Surrey, UK

Despite advances in the treatment of cancer, the death rate remains high. In 2005, more than 153 000 people died from the disease in the United Kingdom (http://www.info.cancerresearchuk.org/cancerstats/mortality/?a=5441); hence anticancer therapies with novel mechanisms of actions are urgently needed. Gene therapy is one promising candidate. Conventional cancer treatments often lack selectivity for tumour cells, leading to toxicity. By contrast, gene therapy allows for the targeting of anticancer agents to the malignant cells, thus minimising damage to normal tissues. An advantage of cancer gene therapy is that it does not require long-term transgene expression, unlike gene therapy for many non-cancer diseases. This is one reason why two-thirds of all gene therapy trials so far have focused on the treatment of cancer (www.wiley.co.uk/genmed/clinical). However, the initial enthusiasm for cancer gene therapy has been dampened by an increasing number of obstacles that need to be overcome before it can be used routinely in the clinic. Cancer gene therapy has reached a critical stage and the editors of *Gene Therapy for Cancer* have identified the need for this book that covers the current technologies of cancer gene therapy as well as the difficult task of applying these in clinical trials.

The book has 67 contributors, mostly based in the United States. It contains over 450 pages of text, divided into three parts: (1) vectors used in gene therapy against cancer, (2) targets and specific approaches for the therapy of cancer and (3) clinical applications of cancer gene therapy. These sections are subdivided into 25 chapters, each of which is written by well-recognised experts on the topic, and prefaced by a summary of the chapter and a list of key words. *Gene Therapy for cancer* includes references up to and including 2004. This reflects the period of time that it takes to complete a multi-authored book. One criticism is that as cancer gene therapy is a rapidly advancing technology, the developments of the last 3 years in this field are absent. The index is extensive, making it easy to find specific information. The book also benefits through its helpful tables, drawings and illustrations.

The first part of the book gives a good overview of the vector systems that have been developed for cancer gene therapy, describing their respective characteristics and production methods. Nonreplicating and oncolytic adenoviral vectors are discussed in chapter 1, followed by a chapter that describes the immunogenicity of these vectors and the pathways of adenoviral infection. Chapter 3 deals with lentiviral and retroviral vector systems (both replicating and nonreplicating), with emphasis on safety considerations. Vaccinia and pox virus vectors are discussed in chapter 4, while chapter 5 describes the development of herpesvirus vectors for clinical applications. Chapter 6 addresses the different types of alphavirus vectors (replication-deficient, replicating and DNA-based). In chapter 7, vesicular stomatitis virus and other RNA viruses are discussed (reovirus, measles, Newcastle disease virus, influenza virus and poliovirus). There is also a chapter on parvoviral vectors (autonomous parvoviruses and adeno-associated virus; chapter 8), before the first part of *Gene Therapy for Cancer* concludes with an overview over nonviral gene delivery systems (chapter 9).

The second part of the book introduces a range of different strategies and targets for cancer gene therapy. It begins with a review of oncogenes, tumour suppressor and apoptosis-inducing genes, and their applications in cancer gene therapy (chapter 10). Chapter 11 then describes gene-silencing approaches, focusing on RNA interference. There are also three chapters dealing with oncolytic viruses; strategies for the re-targeting of adenoviral vectors to tumour cells are discussed in chapter 12, while chapters 13 and 19 discuss oncolytic Herpes simplex vectors and the adenovirus Δ24, respectively. Some of the topics that are discussed in these three chapters have already been covered in the first part of the book and are repetitive. In chapter 14, cell-based and active vaccination strategies to deliver cytokine genes are discussed. Chapter 15 describes combination treatments with cancer gene therapy and conventional radiotherapy, followed by a chapter addressing gene transfer strategies for haematopoietic stem cells. Nonviral gene delivery for genetic vaccines is reviewed in chapter 17 and finally, approaches to inhibit tumour angiogenesis and lymphangiogenesis are described in chapter 18. It is notable that there is comparatively little discussion of approaches that utilise prodrug-activating enzymes in the second part of the book. The field of cancer gene therapy is vast and it impossible to consider every strategy, but a more thorough overview could have been achieved by devoting a whole chapter to this topic.

In the third and last part, the book focuses on cancer gene therapies that have reached the clinical testing stage. The chapters in this section are authored by experts on clinical trials who discuss the difficulties of translating preclinical findings into clinical results and present ways to overcome some of these challenges. Since the approval of the first clinical cancer gene therapy trial in 1989, over 800 trials have been carried out worldwide (www.wiley.co.uk/genmed/clinical). Chapter 20 provides a comprehensive overview of the outcome of these studies, discussing issues such as efficacy, side effects and response indicators. This chapter also gives an insight into the future directions in the field. Chapters 21 and 24 both describe preclinical and clinical data for E1A gene therapy. In chapter 22, the clinical experience with the oncolytic Newcastle disease virus PV701 is reviewed, and chapter 23 discusses mda-7 (IL-24) gene transfer, focussing strongly on preclinical data. The last chapter of the book (chapter 25, written by two of the editors) summarises and reviews all of the steps from the preclinical phase to clinical trial completion; although the regulatory and financial considerations in this chapter will appeal mainly to researchers in the United States, they are not without relevance in Europe.

In summary, *Gene Therapy for Cancer* provides a good overview of currently available techniques for cancer gene therapy and their limitations and potential for clinical applications. The book is a useful addition to the library of both researchers and clinicians working in the field.

